# Spatio-temporal dynamics of resting-state brain networks are associated with migraine disability

**DOI:** 10.1186/s10194-023-01551-y

**Published:** 2023-02-20

**Authors:** Yan Zhou, Liusheng Gong, Yushu Yang, Linjie Tan, Lili Ruan, Xiu Chen, Hua Luo, Jianghai Ruan

**Affiliations:** 1 Department of Neurology, Jianyang People’s Hospital, Jianyang, 641400 China; 2 Department of Neurology, The Affiliated Hospital of Southwest Medical University, Luzhou, 646000 China; 3 Laboratory of Neurological Diseases and Brain Function, Luzhou, 646000 China

**Keywords:** Migraine, EEG microstate, fMRI, Functional connection, Resting state, Brain network

## Abstract

**Objective:**

The changes in resting-state functional networks and their correlations with clinical traits remain to be clarified in migraine. Here we aim to investigate the brain spatio-temporal dynamics of resting-state networks and their possible correlations with the clinical traits in migraine.

**Methods:**

Twenty Four migraine patients without aura and 26 healthy controls (HC) were enrolled. Each included subject underwent a resting-state EEG and echo planar imaging examination. The disability of migraine patients was evaluated by Migraine Disability Assessment (MIDAS). After data acquisition, EEG microstates (Ms) combining functional connectivity (FC) analysis based on Schafer 400-seven network atlas were performed. Then, the correlation between obtained parameters and clinical traits was investigated.

**Results:**

Compared with HC group, the brain temporal dynamics depicted by microstates showed significantly increased activity in functional networks involving MsB and decreased activity in functional networks involving MsD; The spatial dynamics were featured by decreased intra-network FC within the executive control network( ECN) and inter-network FC between dorsal attention network (DAN) and ECN (*P* < 0.05); Moreover, correlation analysis showed that the MIDAS score was positively correlated with the coverage and duration of MsC, and negatively correlated with the occurrence of MsA; The FC within default mode network (DMN), and the inter-FC of ECN- visual network (VN), ECN- limbic network, VN-limbic network was negatively correlated with MIDAS. However, the FC of DMN-ECN was positively correlated with MIDAS; Furthermore, significant interactions between the temporal and spatial dynamics were also obtained.

**Conclusions:**

Our study confirmed the notion that altered spatio-temporal dynamics exist in migraine patients during resting-state. And the temporal dynamics, the spatial changes and the clinical traits such as migraine disability interact with each other. The spatio-temporal dynamics obtained from EEG microstate and fMRI FC analyses may be potential biomarkers for migraine and with a huge potential to change future clinical practice in migraine.

**Supplementary Information:**

The online version contains supplementary material available at 10.1186/s10194-023-01551-y.

## Introduction

Migraine is a functional brain disease [1]. Repeated pain brings a lot of trouble to people's lives, in addition to the common hypersensitivity to sound and light stimulation, autonomic nerve changes, mood changes [2], and even found to lead to attention, memory, and other cognitive disorders [3, 4]. It is the second leading cause of disability in the world [5]. Most of the studies on the pathophysiology of migraine are based on electroencephalogram (EEG) and functional magnetic resonance imaging (fMRI) to analyze the changes in functional connectivity (FC) of the brain networks [6].

EEG microstate analysis can evaluate the functional state of the brain in the sub-second time scale [7], with very high temporal resolution. And extracting and analyzing the BOLD signals in the network nodes constructed by fMRI to achieve FC analysis of brain networks [8] has very high spatial resolution. The temporal process of EEG microstate is associated with the corresponding resting state fMRI (RS-fMRI) network in space [8], and the four microstates correspond to auditory network(AuN), visual network(VN), salience network(SN) and dorsal attention network(DAN) respectively [9, 10]. The joint analysis of EEG microstate and fMRI can reveal the spatio-temporal dynamics of human brain information processing [6], reflect related or identical potential physiological processes on two scales [11], and complement each other in resting state network(RSN) research [12]. At present, it has been found that the functional connections of Alzheimer's disease [8], Lewy body dementia [13]and Autism spectrum disorder [6] have different spatial and temporal manifestations compared with normal healthy subjects.

Previous studies have reported that migraine patients have abnormal FC, including default mode network(DMN) [14], sensorimotor network(SMN) [15], central executive network(CEN)and salience network(SN) [16]. Most of these anomalies are analyzed from the perspective of a single spatial change, while the complex large-scale networks of the human brain are reorganized in different time and space. The normal cooperation and information transmission between networks maintain people's sensory function, cognitive function, and the ability to deal with complex tasks. Various clinical manifestations of migraine may be the result of abnormal brain network connections such as vision, hearing, pain and emotional processing.

These studies partially suggest the brain functional network pathological mechanism involved in migraine. However, our understanding of migraine pathophysiology is still rudimentary. And studies of brain network changes in migraine by combination of EEG and fMRI have been rarely reported. Therefore, here we described the changes of neuronal activity and hemodynamics of migraine from two scales of time and space, and explored the functional network changes in migraine and their correlations with clinical traits to deepen the understanding of the pathophysiological mechanism of migraine.

## Materials and methods

### Participants

The subjects in this study were from June 2020 to May 2022 in the August of Neurology of Jianyang people's Hospital who were diagnosed with migraine without aura according to the third edition of International Classification of headache Diseases [17]and were in the inter-attack period. Inclusion criteria:1. Age: 18–60 years old;2.in the interictal period (no seizures within 48 h before and after the examination [18]); 3. attack frequency less than 15 times per month. Exclusion criteria: 1.secondary headache caused by cerebral hemorrhage, cerebral infarction, aneurysm, intracranial space occupying, arteriovenous malformation and other types of primary headache; 2.complicated with autoimmune diseases or some serious chronic diseases such as heart, liver, renal insufficiency, chronic obstructive pulmonary disease;3.have suffered from mental or nervous system diseases (such as epilepsy, dizziness, cerebral infarction, cerebral hemorrhage, brain tumor, etc.);4.alcohol or drug abusers; 5. subjects with abnormal EEG and MRI results.

In addition, healthy subjects with gender and age matched were recruited as the healthy control (HC) group. The HC group had no history of neurological or psychiatric disorders, no history of head trauma, and no history of migraines. All subjects were right-handed and signed informed consent. This study was approved by the Ethics Committee of Jianyang people's Hospital. The volunteers were informed of the whole experimental procedure.

### Scale evaluation

In this study, all subjects of two groups were assessed by a neurologist and a neuropsychologist. Anxiety and depression were evaluated using the Self-rating Anxiety Scale (SAS) [19] and Self-rating Depression Scale (SDS) [20]. Patients with a score ≥ 50 at SAS were considered anxiety [19], while a score ≥ 53 at SDS suggests the presence of depressed [20]. And the Mini-mental Status Examination (MMSE) was evaluated to test cognitive function of subjects [21] too. In addition, the migraine patients’ disability was quantified using the Migraine Disability Assessment (MIDAS) [22]. 0–5 indicates mild disability or no disability; 6–10 indicates mild disability; 11–20 indicates moderate disability; ≥ 21 indicates severe disability [23].

### Data acquisition

#### EEG data collection

All subjects collected 16-lead (Fp1, Fp2, F3, F4, C3, C4, P3, P4, O1, O2, F7, F8, T3, T4, T5, T6) EEG data for 20 min while keeping quiet and closing their eyes in the same dark room with suitable temperature. 500 Hz electroencephalograph (NT9200, Beijing Xintuo Co., Ltd.) was used to place electrodes according to the international 10–20 system (American Clinical Neurophysiology Society). The resistance of all electrodes is reduced to less than 5KΩ. During the examinations, all participants were told to keep mind-wandering with eyes closed.

#### fMRI data collection

The fMRI data acquisition was performed with a 3.0 Tesla (Philips 3.0 T Medical Systems, Nederland) MRI scanner using Blood oxygenation level dependent (BOLD) echo-planar imaging (EPI) sequence (Field of view (FOV) = 240 × 240 mm^2^, Time repetition (TR) = 2000 ms, Time point = 180, Voxel size = 1.67 mm × 1.67 mm × 3.0 mm, Flip angle = 90°, Time echo = 35 ms, Matrix = 144 × 144, 48 slices, Layer thickness / gap = 3 mm/0.0 mm). Sponge pads and earplugs were employed to control head motion and reduce the influence of scanner noise during the scanning. All participants were told to hold still, keep their eyes closed and mind-wandering during the examinations.

### Data preprocessing

#### EEG data preprocessing

The EEG data of each subject were exported to the standard format European Data Format (.EDF) and numbered. The derived data include 16 leads: Fp1, Fp2, F3, F4, F7, F8, C3, C4, T3, T4, T5, T6, P3, P4, O1, O2. Each subject's EEG data was imported into EEGlab(v14.1.1, http://sccn.ucsd.edu/) based on Matlab (R2014a MathWorks, US) and processed in a pipeline. filtering the data with a bandpass of 1–45 Hz. removal and 3D spline interpolation of bad channels. The number of interpolated electrodes was less than 2 for each participant. Eye movement and EMG interference are removed automatically using an EEGlab plugin-in automatic artifacts removal (AAR) (http://germangh.com). re-referencing to the average lead of the reference electrode. Then, five 10 s segments of EEG data with eyes closed, awake and without obvious interference were selected. the preprocessed data was converted to text file format. Each subject finally got EEG preprocessing data containing five text files for follow-up analysis.

#### fMRI data preprocessing

The origin data were export in Digital Imaging and Communications in Medicine (DICOM) format. The Matlab (R2014a MathWorks, US) toolbox Statistical Parametric Mapping tool(SPM12) [24] (http://www.fil.ion.ucl.ac.uk/spm/software/spm12) and Data Processing Assistant for Resting-State fMRI v2.3 (DPARSF,www.restfmri.net) [23] were used in the. The preprocessing pipeline included following steps: converting the data from DICOM format to NIfTI format; removing the first four volumes; slice-timing correction; motion realignment; spatial normalization using the EPI template to the stereotactic space of the Montreal Neurological Institute (MNI) with a voxel size of 3 × 3 × 3 mm; Nuisance covariates including head motion parameters and linear trends were regressed out from the signals; Motion-corrupted volumes were interpolated with neighbor volumes. Gaussian spatial smoothing was performed (FWHM = 4 mm); Finally, we performed temporal bandpass filtering (0.01–0.1 Hz) across time series. Those data with mean Jenkinson framewise displacement (FD) larger than or equal to 0.2 would not be further analyzed.

### EEG Microstate and fMRI FC analyses

#### Microstate analysis

In this study, the microstate analysis module in LORETA-Key (www.uzh.ch/keyinst/loreta) analysis software is used to complete the microstate analysis. According to the commonly used calculation method of microstate method in the current research [25–27], the electric field intensity of brain signal at each time point is firstly calculated using the following formula, which is expressed as global field power (GFP).

1 $$\boldsymbol{ }\boldsymbol{ }\boldsymbol{ }\boldsymbol{ }\boldsymbol{ }\boldsymbol{ }\boldsymbol{ }\boldsymbol{ }\boldsymbol{ }\boldsymbol{ }\boldsymbol{ }\boldsymbol{ }\boldsymbol{ }\boldsymbol{ }\boldsymbol{ }\boldsymbol{ }\boldsymbol{ }\boldsymbol{ }\boldsymbol{ }\boldsymbol{ }\boldsymbol{ }\boldsymbol{ }\boldsymbol{ }\boldsymbol{ }\boldsymbol{ }\boldsymbol{ }\boldsymbol{ }\boldsymbol{ }\boldsymbol{ }\boldsymbol{ }{\varvec{G}}{\varvec{F}}{\varvec{P}}({\varvec{t}})=\sqrt{\frac{{\Sigma }_{i=1}^{n}{\left[vi\left(t\right)-\overline{v }\left(t\right)\right]}^{2}}{n}}$$

n means the number of leads. vi (t) and v(t) indicates for the potential of the corresponding i^th^ electrode, the mean potential of the 16 leads at time t, respectively. Thus, the value of GFP means the changes in the potential among electrodes in each given time. Then, the topographic map of the moment of the peak of GFP is analyzed by k-means clustering method [12]. Thus, the main terrain template of functional microstate is determined [28].Then the large residual noise is removed by the cross-verification standard, and the optimal number of micro-states is determined. Finally, the spatial correlation between the best micro-state topographic map and all the initial topographic maps is analyzed. Find out the micro-state topographic map with minimum global topographic map difference global map dissimilarity (GMD).

Previous studies have found that four types of micro-state topographic maps for resting-state EEG showed a good stability and repeatability [29–31]. Therefore, we set the number of microstates at ‘4’.The changes of brain functional state were described by four microstate parameters: mean duration, occurrence per second, coverage and transition probability [12, 32].

#### Sources of microstates

The inverse eLORETA method of the Loreta Key software was used to reverse the transformation of each microstate, and the MNI template [33]was used for calculation. The eLORETA files of each microstate were obtained for source location analysis [34]to find the brain regions synchronized with each microstate and their corresponding neural networks [34]. The eLORETA method is used to locate EEG data from multiple distributed cortical sources in three-dimensional space [35]. Compared with sLORETA, the localization error is smaller, and it can also provide accurate location in the case of better positioning effect, clearer image quality and structured biological noise [36]. It was first proposed by Pascual-Marqui et al. in 1994 [37]. It is widely used in a variety of EEG data research [38–40]. For specific mathematical algorithms, please see [36].

#### Large-Scale functional connectivity analysis

After the fMRI data preprocessing, the functional brain network was constructed by MATLAB toolbox DPABI [23]. According to the schaefer-400 template [41], the cortical and subcortical regions were divided into 400 functional regions of interest (ROI) as extraction time nodes. These ROIs were composed of 7 brain networks including DMN, SMN, dorsal attention network (DAN), ventral attention network (VAN), executive control network (ECN), Visual network (VN), limbic network [41]. The average time series of each node was extracted. Then, the pairwise functional connectivity (FC) between time series was estimated by calculating the linear Pearson correlation coefficient. Then, the intra and internetworks of FC were calculated by using the mean value of edges in the corresponding subnetwork.

#### Correlation analysis

In order to understand the correlation between microstate changes, brain network changes and migraine clinic traits, Pearson correlation coefficient were calculated between parameters involving EEG microstate, MIDAS score, intra- and inter-network FC after controlled for age and sex as covariables. We took gender and age as covariables to analyze the correlation between EEG microstate parameters and MIDAS, and took gender, age and FD as covariables to evaluate the correlation between intra- and inter-network changes and MIDAS. In addition, we analyzed the correlation between microstate parameters and brain network to further observe the relationship between EEG and fMRI brain functional interactions.

#### Subgroup analysis

Considering that disease duration, attack frequency, pain sides, and medication during attacks may have impacts on brain functional networks, we divided the migraine patients into subgroups based on these conditions and compared the parameters including inter- and intra-network FC, microstate parameters of the subgroups.

#### Statistical evaluations

A generalized linear model (GLM) with age and sex (and mean Jenkinson FD for fMRI data) as covariates was used to exclude the potential influences of these factors on the results of group comparisons and correlation analysis. A two-sample *t* test with False Discovery Rate (FDR) correction was used to compare the group or sub-group differences in parameters including microstates and FC. Permutation test (iteration = 10 000) was used to further confirm the results of comparisons. Cohen’s *d* value was calculated to depict the effect size between two variables. For the correlation analysis, Pearson correlation coefficient r and their 95% confidence interval were calculated.

## Results

### Demographic and clinical data results

According to the inclusion criteria and exclusion criteria, 24 patients with migraine and 26 healthy controls were included in this study. Table 1 summarized the demographic characteristics and clinical data of all subjects. We found that there were no significant differences in age, sex, time of education, SAS scores, SDS scores, MMSE scores and the Jenkinson FD between the two groups(*P* > 0.05). The individual characteristics of the migraine patients are shown in S.Table 1.

**Table 1:** Demographics and scales of migraine patient and control groups^a^

	Patient	Control	*χ2/t*	*P*
Sample size	24	26	\	\
Age(years)^c^	34.88 ± 1.95	37.92 ± 2.54	0.94	0.35
Sex (Male/Female)^b^	3/21	5/21	0.42	0.70
Education (years)^c^	13.96 ± 0.51	13.77 ± 0.58	0.24	0.81
Disease duration (years)	7.65 ± 1.34	\	\	\
frequency (attacks per month)	2.79 ± 0.49	\	\	\
SAS^c^	38.6 ± 1.96	34.45 ± 1.40	1.75	0.09
SDS^c^	37.81 ± 1.87	35.38 ± 1.64	0.98	0.33
MMSE^c^	27.29 ± 0.43	28.27 ± 0.38	1.71	0.09
MIDAS	7.18 ± 1.27	\	\	\
FD^c^	0.08 ± 0.01	0.07 ± 0.01	0.72	0.48

^a^The statistical results are expressed as mean ± standard error of mean (Mean ± SEM)

^b^Chi-square test

^c^Two sample *t*-test

*SAS* Self-rating anxiety scale, *SDS* Self-rating depression scale, *MMSE* Mini-mental status examination, *MIDAS* Migraine disability assessment, *FD* Mean Jenkinson framewise displacement

### Microstate analysis

#### Temporal dynamics of EEG microstates

**Fig.1:**
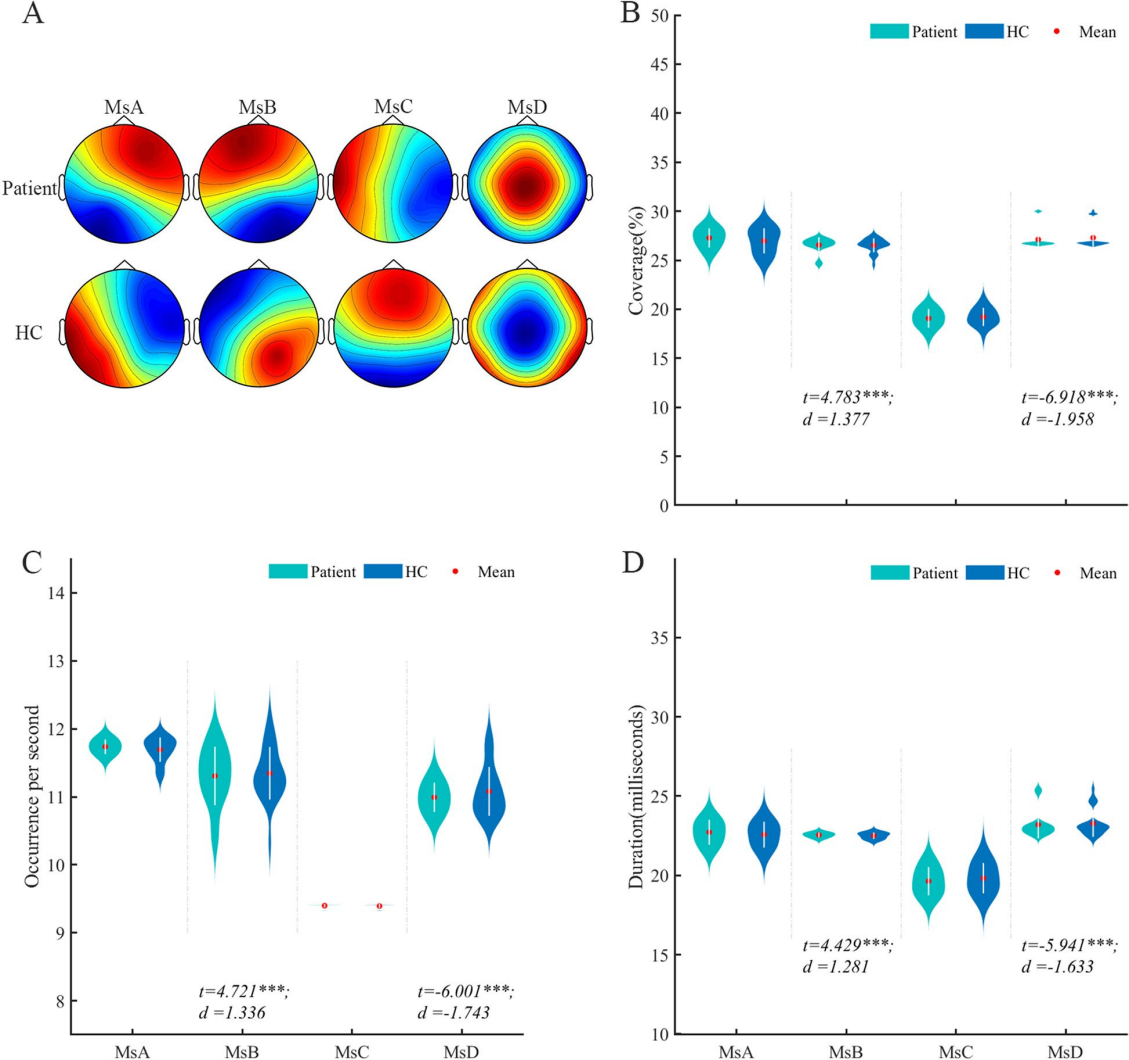
Comparison of four microstates and temporal dynamics between migraine patients and healthy controls. **A**. The four microstates recognized by k-means cluster analysis across subjects in the patient and control groups. **B**. The coverage of the four microstates in each group. **C**. The occurrence per second of the four microstates in each group. **D**. The duration of the four microstates in each group. The comparisons of microstate parameters between patient and HC were assessed by using a generalized linear model (GLM) with age and sex as covariates. And the results have been further confirmed by permutation test (iteration = 10,000) using residuals of microstate parameters after GLM. HC, healthy control; Ms, microstate; *d*, the effect size Cohen’s *d*

Through K-means cluster analysis, migraine patients and healthy controls finally got four microstates, which were marked as A, B, C and D (Fig. 1). By using the method of two-sample t-test, the microstate coverage, occurrence per second, duration, and microstate transition probabilities between the patient group and the control group were compared.

**Fig.2:**
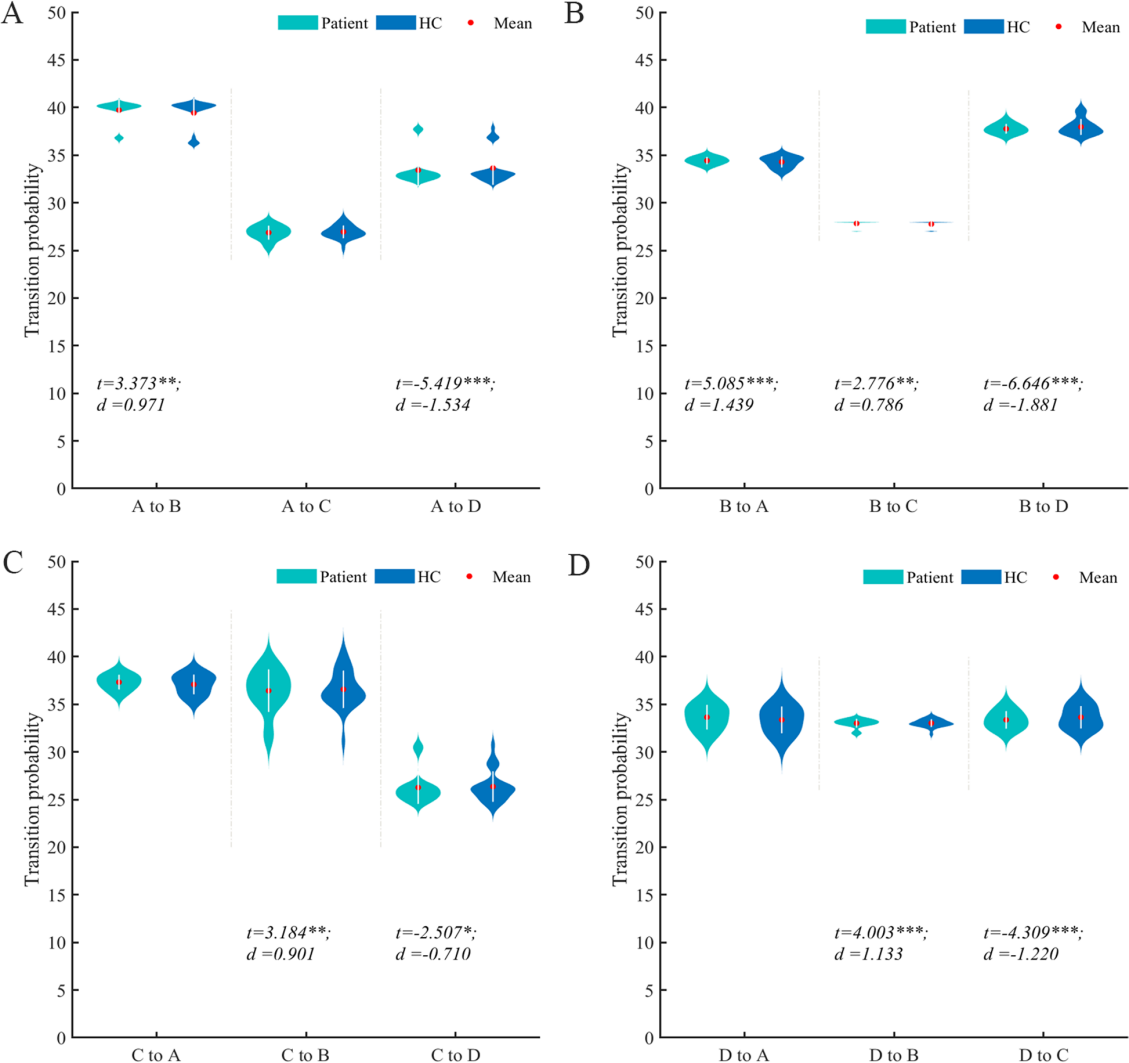
Comparison of Markov chain transition probabilities of the four microstates in patient and control groups. **A**. The transition probabilities from microstate A to other microstates; **B**. The transition probabilities from microstate B to other microstates. **C**. The transition probabilities from microstate C to other microstates. **D**. The transition probabilities from microstate D to other microstates. The transition probabilities from one microstate to another were calculated using Markov chains and assessed by using GLM with age and sex as covariates. The results have been further confirmed by permutation test (iteration = 10,000) using residuals of microstate parameters after GLM. HC, healthy control; *d*, the effect size Cohen’s *d*. ***P < 0.001, **P < 0.01, *P < 0.05

Compared with the control group, the coverage (*P* < 0.001, *t* = 4.783, Cohen’s *d* = 1.377), occurrence per second (*P* < 0.001,* t* = 4.721, Cohen’s *d* = 1.336), duration (*P* < 0.001, *t* = 4.429, Cohen’s *d* = 1.281) of microstate B (MsB) increased in migraine patients, while the coverage (*P* < 0.001, *t* = -6.981, Cohen’s *d* = -1.958), occurrence per second (*P* < 0.001,* t* = -6.001, Cohen’s *d* = -1.743), duration (*P* < 0.001, *t* = -5.941, Cohen’s *d* = -1.633) of microstate D (MsD) decreased compared with the healthy group. There was no significant difference in the change of microstate A (MsA) and microstate C (MsC) between the two groups(Fig. 1). Compared with the HC group, the bidirectional conversion rate between MsA and MsB, MsB and MsC were increased, the bidirectional transition probability between MsC and MsD decreased, the transition from MsB to MsD decreased in migraine group, while MsD transferred more easily to MsB (*P* < 0.05). There was no significant change in the transfer probability between MsA and MsC (*P* > 0.05) (Fig. 2).

#### Source localization of microstates

**Fig.3:**
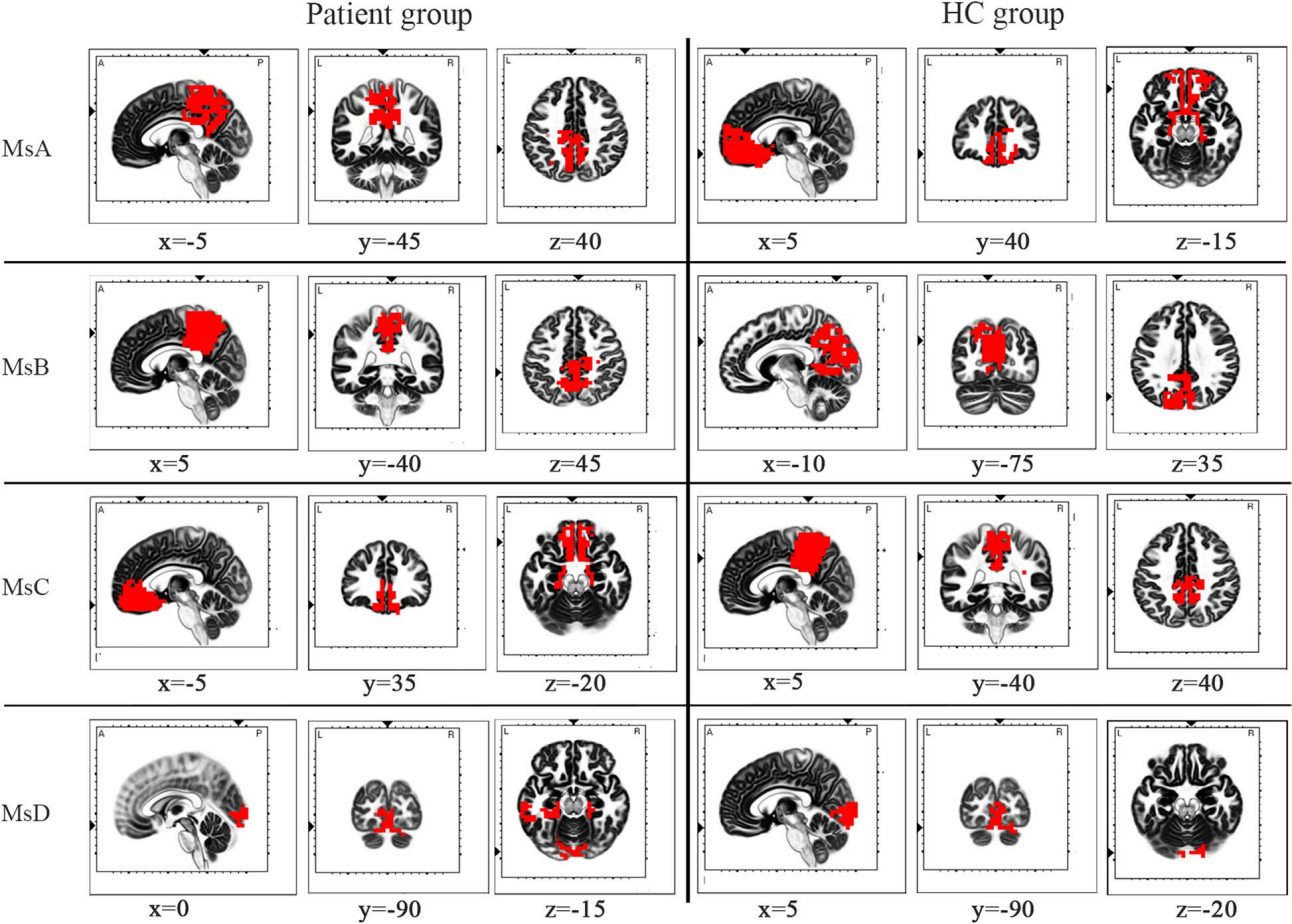
The eLORETA images of corresponding microstates shown in MNI brain for patient and control groups. The red color indicates the maximum current density (> 75%). The small triangles along the coordinate axes indicated the maximum electric neuronal activity and the corresponding peak coordinates were displayed in lower part of each intercepted image. The corresponding peak coordinates are displayed above each captured image. Microstates A to D in the patient group were in Cingulate Gyrus (BA31), Precuneus (BA7), Medial Frontal Gyrus (BA11), Lingual Gyrus (BA18). Microstates A to D in the control group were in Medial Frontal Gyrus (BA11), Precuneus (BA7), Cingulate Gyrus (BA31), Lingual Gyrus (BA18)

The cortical localization of the electrical activity of neurons in four microstates in migraine patients and healthy controls was performed by eLORETA. The specific localization map is shown in Fig. 3.

The localization according to the MNI peak coordinates is as follows: migraine patient group (from MsA to MsD): Cingulate Gyrus (BA31), Precuneus (BA7), Medial Frontal Gyrus (BA11), Lingual Gyrus (BA18); healthy controls group (from MsA to MsD): Medial Frontal Gyrus (BA11), Precuneus (BA7), Cingulate Gyrus (BA31), Lingual Gyrus (BA18).

#### The changes of intra- and inter-network FC of two groups

**Fig.4:**
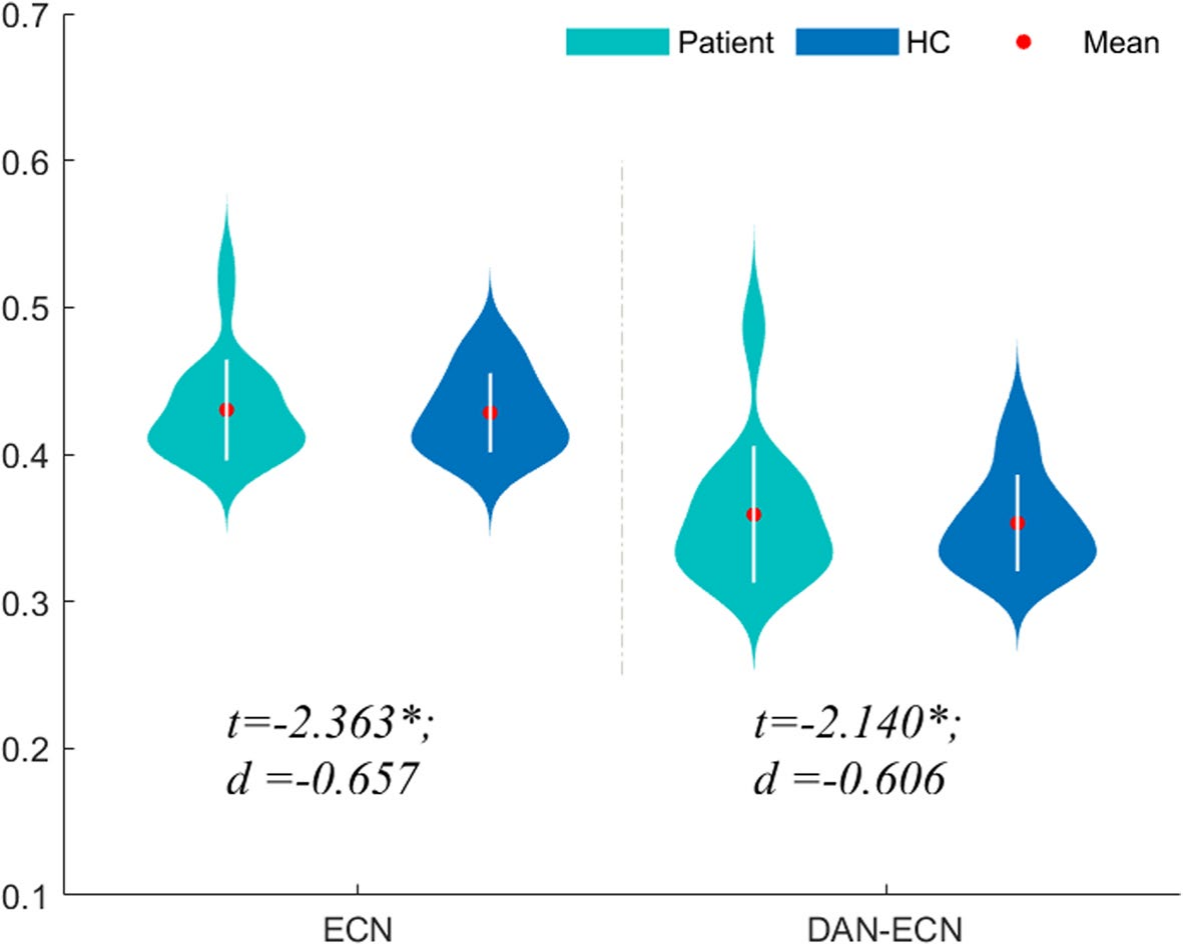
Subnetwork connections of fMRI in intra-network and inter-network comparison between patient group and HC group. The comparisons between two groups were assessed by using two-simple *t* test with age, sex and FD as covariates. DAN, dorsal attention network; ECN, executive control network; HC, healthy control; d, the effect size Cohen’s d. *P < 0.05

After preprocessing the fMRI data, the time series of schaefer 400 ROIs in 7 networks of the subjects were extracted. The average intra- and inter-network connections of the functional networks of the patient group and the HC group were calculated. Compared with HC group, the connection in the ECN in the migraine group was weaker (*P* < 0.05, *t* = -2.363, *d* = -0.657), and the functional connection between the dorsal attention network (DAN) and the ECN was decreased (*P* < 0.05, *t* = -5.941, *d* = -1.633) (Fig. 4). The FCs within and between other networks did not change significantly (*P* > 0.05).

### Correlation analysis

#### Correlation between Ms and MIDAS

**Fig.5:**
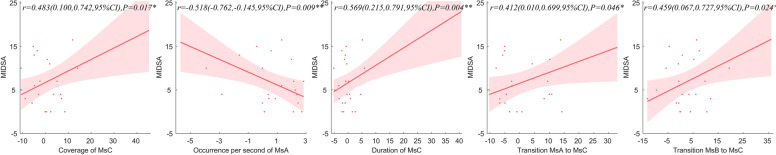
The correlation between microstate parameters and MIDAS scores of patient group. **A**. The correlation between MIDAS and coverage of MsC. **B**. The correlation between MIDAS and occurrence per second of MsA. **C**. The correlation between MIDAS and duration of MsC. **D**. The correlation between MIDAS and transition MsA to MsC. **E**. The correlation between MIDAS and transition MsB to MsC. The correlation was analyzed by the residuals of microstate parameters and MIDAS with age and sex as covariates. Ms, microstate, CI, confidence intervals; **P < 0.01, *P < 0.05

MIDAS was positively correlated with the coverage (*r* = 0.483) and duration (*r* = 0.569) of MsC in the patient group, negatively correlated with the occurrence per second of MsA (*r* = 0.518), and positively correlated with the transfer probability of MsA (*r* = 0.412) and MsB (*r* = 0.459) to MsC (Fig. 5).

#### Correlation between intra-, inter-network FC and MIDAS

**Fig.6:**
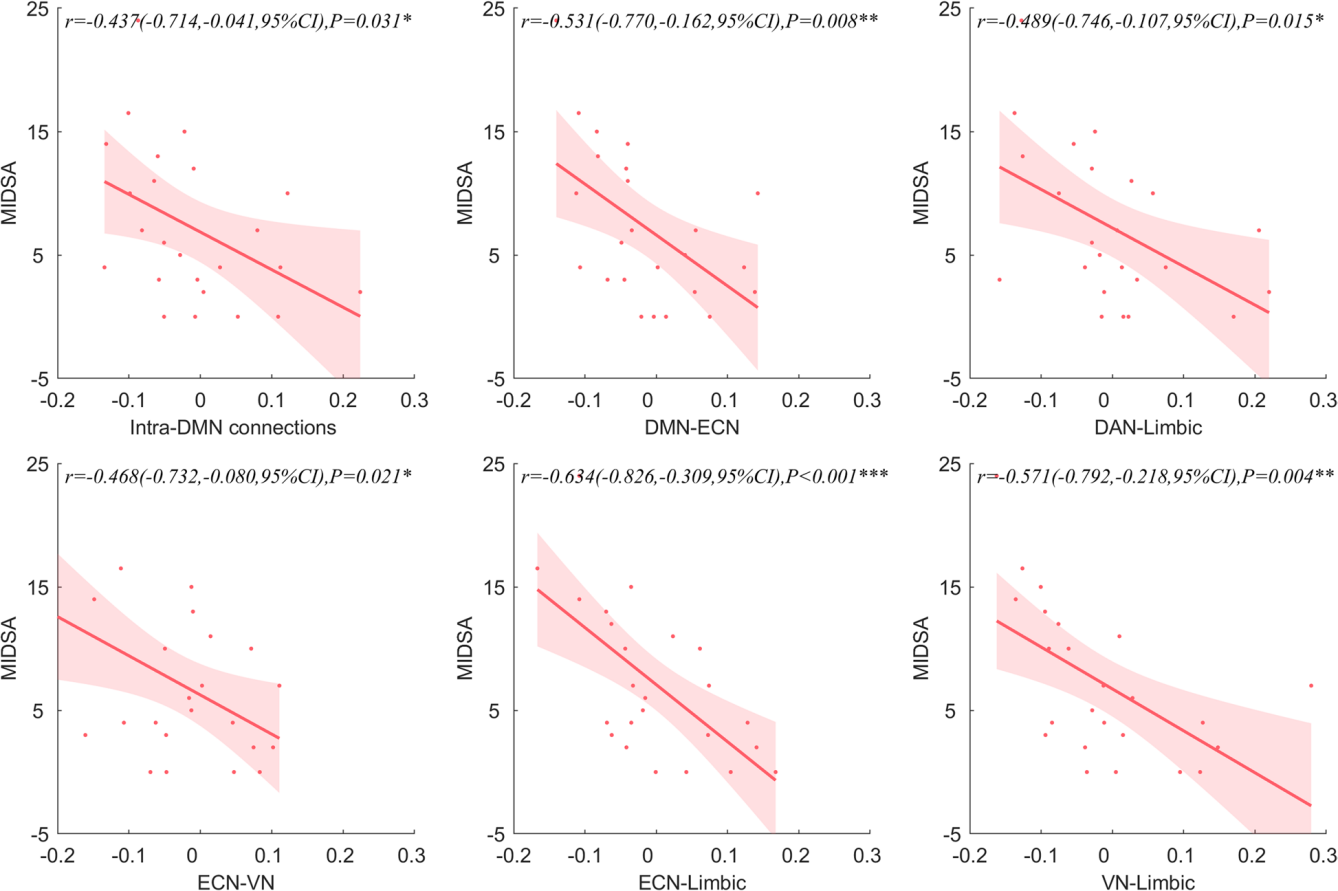
The correlation between Intra- and Inter-network FC and MIDAS of patient group. **A**. The correlation between MIDAS and intra-DMN connection. **B**. The correlation between MIDAS and the FC of DMN and ECN. **C**. The correlation between MIDAS and the FC of DAN and limbic network. **D**. The correlation between MIDAS and the FC of ECN and VN. **E**. The correlation between MIDAS and the FC of ECN and limbic network. **F**. The correlation between MIDAS and the FC of VN and limbic network. The correlation was analyzed by the residuals of intra-network connection and inter-network connection time series and MIDAS with age, sex and FD as covariates. DMN, default mode network; DAN, dorsal attention network; VN, visual network; ECN, executive control network, CI, confidence intervals; ***P < 0.001, **P < 0.01, *P < 0.05

It was found that there was a negative correlation between the connection intensity of intra-DMN and MIDAS (*r* = 0.437). The connection between DMN and ECN was positively correlated with MIDAS (*r* = 0.531). The FC between DAN and limbic network (*r* = -0.489), ECN and VN (*r* =—0.468), ECN and limbic network (*r* = -0.634), VN and limbic network (*r* = -0.571) were negatively correlated with MIDAS. (Fig. 6).

#### Correlation between intra- and internetwork FC and Ms of patient group

**Fig.7:**
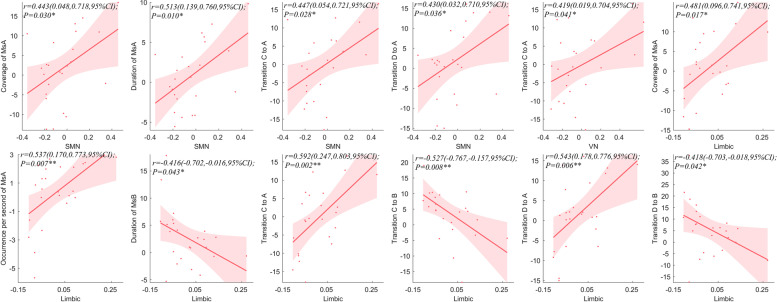
The correlation between Intra- and Internetwork FC and microstate parameters of patient group. **A**. The correlation between SMN and coverage of MsA. **B**. The correlation between SMN and duration of MsA. **C**. The correlation between SMN and transition probability from MsC to MsA. **D**. The correlation between SMN and transition probability from MsD to MsA. **E**. The correlation between VN and transition probability from MsC to MsA. **F**. The correlation between limbic and coverage of MsA. **G**. The correlation between limbic network and occurrence per second of MsA. H. The correlation between limbic network and duration of MsB. I. The correlation between limbic network and transition probability from MsC to MsA. J. The correlation between limbic network and transition probability from MsC to MsB. K. The correlation between limbic network and transition probability from MsD to MsA. L. The correlation between limbic and transition probability from MsD to MsB. The correlation was analyzed by the residuals of intra-network connection and inter-network connection time series and microstate parameters with age, sex and FD as covariates. Ms, microstate; SMN, sensory-motor network; VN, visual network, CI, confidence intervals; *P < 0.05

By analyzing the correlation between the average intra-network and inter-network connections and microstate parameters of migraine, it was found that SMN was positively correlated with the coverage (*r* = 0.443,) and duration (*r* = 0.513) of MsA, and with the probability of transfer from MsC (*r* = 0.447) and MsD (*r* = 0.430) to MsA. VN was positively correlated with the transition probability from MsC to MsA (*r* = 0.419). The limbic network was positively correlated with the coverage (*r* = 0.481) and occurrence per second (*r* = 0.537) of MsA, negatively correlated with the duration of MsB (*r* = -0.416), positively correlated with the probability of transfer from MsC (*r* = 0.592) and MsD (*r* = 0.543) to MsA, and negatively correlated with the probability of transfer from MsC (*r* =—0.527) and MsD (*r* =—0.418) to MsB (Fig. 7). There was no above correlation between microstate parameters and intra-network and inter-network in HC group (S.Fig. 1).

#### Subgroup analysis

We found the parameters involving inter- and intra-network FC and microstates showed no differences between the two subgroups divided by disease durations (disease durations > 5 years compared with disease durations ≤ 5 years). However, when we divided the subgroups by the attack frequency, we noted that compared with the patients with attacks less than or equal to 2 per month, those patients had attacks larger than 2 per month showed significantly declined inter-network (SMN-DAN, SMN-VN, DAN-VN) FC and intra-network (SMN, DAN, VN) FC(S.Fig. 2, S.Fig. 3).

The effect of pain sides on these parameters was also investigated. Compared with the migraine patients with left-side pain, the migraine patients with bilateral headaches showed decreased intra-network connectivity of DMN (S.Fig. 4). Finally, we divided the migraine patients into two groups by their medication status during attacks. Compared with the migraine patients taking paracetamol during attacks, those migraine patients taking ibuprofen showed decreased MsA occurrence per second, and decreased inter-network FC involving DMN-VAN, VAN-limbic and VN-limbic (S.Fig. 5, S.Fig. 6). The other parameters we did not mention showed no differences in these comparisons.

## Discussion

In this study, we found that the brain networks of migraine patients changed through EEG microstate analysis and fMRI FC analysis, and there was a spatio-temporal correlation. The interaction and reorganization of multiple brain networks in different time and space is the possible pathophysiological mechanism of migraine visual processing disorders, pain processing, emotional changes and other clinical manifestations.

It is well known that MsB can reflect the static state VN and its functional state [9, 42]. In this study, we found that the duration, transition probability and coverage of MsB in the patient group were significantly higher than those in the HC group, which confirmed the abnormal function of VN in migraine patients without aura [43]. This change of MsB is related to the increase of cortical and subcortical nerve activity in migraine patients without aura after visual stimulation [29], which is the possible mechanism that migraine patients are prone to photophobia. Coppola G et al. found abnormal FC of visual primary sensory cortex network in migraine patients based on fMRI study [44]. Although we did not find abnormal VN through fMRI FC, the change of MsB indicates that this may be the reflection of abnormal neural activity and network abnormality in visual cortex on different time and spatial scales. After all, EEG microstate gives priority to fMRI by two orders of magnitude in terms of time process [45]. The change from fast EEG time to slow fMRI space can reflect the same potential physiological process of VN changes in migraine patients without aura from two scales [11]. Whether the inconsistency of the two time course changes in our study is related to the course of disease and pain intensity in the included patient group needs further study.

ECN and DAN have synergistic effects on pain processing [46]. In fact, most resting-state fMRI studies of migraine patients have found the FC reduced between them [47, 48]. However, we noticed that in migraine patients, in addition to the reduction of FC between DAN and ECN, MsD parameters and FC within the ECN also decreased significantly. The resting state DAN is considered to correspond to MsD [9],mainly responsible for identifying sensory stimuli, while ECN is responsible for the cognitive selection of relevant sensory information, stimulus processing, and the choice of action responses such as transfer and inhibition [47]. From a temporal dynamic perspective, the reduced of MsD (DAN) leads to attentional deficits [48], while the decrease of FC within the ECN confirms the longer response time of migraine patients to pain processing from the aspect of spatial dynamics [49]. We believe that the changes of MsD and ECN not only fully explain the time process of the change of network FC in pain management, but also further explain the possible pathophysiological mechanism of ECN and DAN co-processing pain dysfunction from the perspective of time and space. However, whether there are other potential changes in the time-to-space change process of DAN needs to be verified by more research. After all, some studies have reported that the coverage and occurrence of MsD in migraine patients are contrary to our results by EEG microstate analysis [29]. Of course, the possible reason for this difference is also related to the fact that we only included migraine patients during interictal periods.

In migraine patients, the decrease of FC intra-DMN FC and between DMN-ECN was negatively correlated with MIDAS score. The MIDAS score indirectly reflects the intensity of pain based on the impact of pain frequency on life, work, and social interaction [50]. It is suggested that the abnormal DMN FC in migraine patients will further lead to disability with the increase of attack frequency [47]. This may be related to ACC and MFG, the main brain regions of DMN [51, 52]. It is worth noting that we also found changes in these two brain regions in the analysis of microstate source localization with more advantages in time progression. It is agreed that this is mainly due to the dysfunction of pain intensity regulation caused by the abnormal functional connection of DMN [53]. In fact, DMN is also a key part of top-down modulation of sensory function [54], promoting the retrieval and integration of related stimuli [55]. In this study, it was found that there was a positive correlation between DMN and the occurrence of MsB and the transfer probability of MsC to MsB in HC group. When the visual information processing of the patient group was impaired, the wrong visual information would disturb the FC correlation between DMN and VN [56], resulting in the disappearance of the correlation between networks. This is a supplement to most current views on abnormal connections to DMN functions [14, 51]. In addition, we also found that the coverage and duration of MsC corresponding to SN and the transfer probability of MsA and MsB to MsC were positively correlated with MIDAS, which also verified that SN was responsible for regulating headache intensity and attack frequency [43]. In fact, DMN and SN are the main cognitive networks [57]. The correlation between SN and DMN and MIDAS exists in two different temporal dimensions, and this difference may be related to the selective formulation of DMN or ECN by SN based on external input information [58], thereby affecting the cognitive function of migraine.

According to previous studies, the resting-state AuN, VN, SN and DAN corresponding to microstates A, B, C and D [9, 10]. In the study, it is found that the coverage of MsA and the transfer probability of MsC and D to MsA are positively correlated with SMN and limbic network, while the duration of MsB and the transfer probability of MsC and MsD to MsB are negatively correlated with limbic network. The possible reasons for these correlations are: both AuN and SMN belong to low-level perceptual network [59], which is responsible for pain afferent [60]; the correlation between limbic network and AuN is associated with anxiety and depression [61],while the correlation with VN leads to increased pain sensitivity [62]. Migraine patients combine sensory information of auditory, visual, and somatosensory perception and recurrent painful experience to regulate the regions of emotion, sensitivity and attention through different spatial and temporal channels. However, no abnormality of SMN, limbic network and AN representing MsA was found in the results. The correlation between microstate parameters and SMN and limbic network in brain network analysis and the change of transfer probability between microstates indicate that each network mainly affects SMN and limbic network after different coordination of time accumulation effect on sub-second time scale, which leads to the reorganization of each network in spatial mode. It causes dysfunction of migraine in sensory discrimination, emotional and cognitive evaluation. The FC of complex cortical networks is related to the frequency and severity of migraine attacks [63]. The negative correlation between DNA and limbic networks, ECN and VN, ECN and limbic networks, VN and limbic networks and MIDAS indicates that weak functional activity between networks will lead to more headache disabilities [15].

There are limitations in this study. We did not do longitudinal cohort follow-up study. And the relationship between these analytical parameters and the prognosis of migraine patients needs to be further studied after more samples collected.

## Conclusion

In this study, EEG microstate analysis and fMRI brain network analysis were combined to confirm the temporal and spatial dynamic changes of brain network in resting state of migraine and its possible correlation with clinical characteristics. The changes in temporal dynamics depicted by microstates are characterized by increased activity in functional networks involving MsB and decreased activity in functional networks involving MsD; The spatial dynamics are featured by decreased intra-network FC within the executive control network( ECN) and inter-network FC between dorsal attention network (DAN) and ECN; Moreover, the temporal dynamics, the spatial changes and the clinical traits such as migraine disability may interacted with each other. These findings may enable us to identify the potential biomarkers of migraine and provide a possible basis for clinical practice of migraine in the future.

## Supplementary Information

**Additional file 1: Table 1. **The individual characteristics of the migraine patients. **Fig. 1** The correlation between Intra- and Internetwork FC and microstate parameters of HC group. **Fig. 2 **Comparison of intra-network FC between subgroups divided by the attack frequency. **Fig. 3** Comparison of inter-network FC between subgroups divided by the attack frequency. **Fig. 4** Comparison of intra-network FC between subgroups divided by pain sides. **Fig. 5** Comparison of microstate parameters between subgroups divided by the medications used during attacks. **Fig. 6** Comparison of inter-network FC between subgroups divided by the medications used during attacks.
