# Supplementary material for: Spatio-temporal dynamics of resting-state brain networks are associated with migraine disability
Source: J Headache Pain. 2023 Feb 20;24(1):13. doi: 10.1186/s10194-023-01551-y (PMC9940435; doi:10.1186/s10194-023-01551-y)
Supplement: Supplementary file 1 — Additional file 1: Table 1. The individual characteristics of the migraine patients. Fig. 1 The correlation between Intra- and Internetwork FC and microstate parameters of HC group. Fig. 2 Comparison of intra-network FC between subgroups divided by the attack frequency. Fig. 3 Comparison of inter-network FC between subgroups divided by the attack frequency. Fig. 4 Comparison of intra-network FC between subgroups divided by pain sides. Fig. 5 Comparison of microstate parameters between subgroups divided by the medications used during attacks. Fig. 6 Comparison of inter-network FC between subgroups divided by the medications used during attacks. [file 10194_2023_1551_MOESM1_ESM.docx]

**Supplementary materials**

S.Table 1: The individual characteristics of the migraine patients*

*M, male; F, female; B, bilateral; L, left; R right; MIDAS, Migraine Disability Assessment.

| Subject | Age | Sex | Years suffering | Pain hemisphere | Attacks/month | MIDAS | Accompanying symptoms | Medications |
| --- | --- | --- | --- | --- | --- | --- | --- | --- |
| 1 | 48 | F | 2 | L | 4 | 3 | / | paracetamol |
| 2 | 51 | F | 20 | B | 5 | 24 | vomiting | ibuprofen |
| 3 | 20 | F | 5 | L | 3 | 4 | / | paracetamol |
| 4 | 25 | F | 3 | B | 2 | 0 | / | / |
| 5 | 39 | F | 20 | B | 2 | 7 | nausea | ibuprofen |
| 6 | 38 | F | 10 | L | 4 | 10 | vomiting photophobia phonophobia | ibuprofen |
| 7 | 36 | F | 10 | R | 2 | 5 | phonophobia | paracetamol |
| 8 | 26 | F | 4 | B | 2 | 13 | nausea vomiting photophobia phonophobia | ibuprofen |
| 9 | 52 | F | 10 | L | 2 | 7 | nausea vomiting | paracetamol |
| 10 | 24 | F | 2 | L | 2 | 16.5 | nausea | ibuprofen |
| 11 | 29 | M | 1 | B | 1 | 4 | nausea | ibuprofen |
| 12 | 26 | F | 0.5 | L | 1 | 10 | / | / |
| 13 | 27 | M | 2 | B | 2 | 15 | nausea vomiting | ibuprofen |
| 14 | 21 | F | 3 | B | 3 | 3 | / | ibuprofen |
| 15 | 28 | F | 8 | B | 4 | 0 | vomiting | ibuprofen |
| 16 | 29 | F | 10 | R | 2 | 2 | nausea | ibuprofen |
| 17 | 49 | F | 1 | L | 13 | 0 | / | ibuprofen |
| 18 | 37 | M | 6 | B | 2 | 0 | nausea | paracetamol |
| 19 | 38 | F | 1 | L | 3 | 12 | nausea | / |
| 20 | 44 | F | 5 | L | 1 | 4 | phonophobia | ibuprofen |
| 21 | 40 | F | 10 | B | 1 | 11 | nausea vomiting photophobia phonophobia | ibuprofen |
| 22 | 35 | F | 20 | R | 2 | 14 | nausea | paracetamol |
| 23 | 33 | F | 10 | B | 2 | 2 | nausea | ibuprofen |
| 24 | 42 | F | 20 | B | 2 | 6 | nausea | paracetamol |


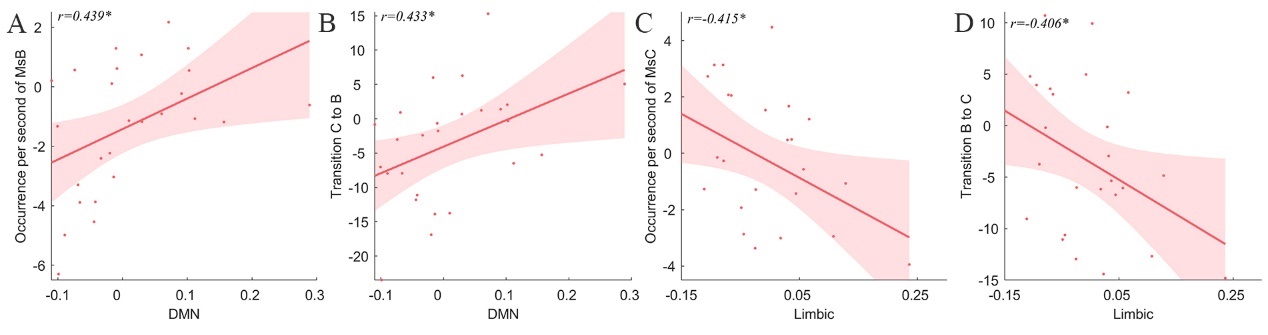


S.Fig.1 The correlation between Intra- and Internetwork FC and microstate parameters of HC group. A. The correlation between DMN and occurrence per second of MsB. B. The correlation between DMN and transition probability MsC to MsB. C. The correlation between limbic network and occurrence per second of MsC. D. The correlation between limbic and transition probability from MsB to MsC. The correlation was analyzed by the residuals of intra-network connection and inter-network connection time series and microstate parameters with age, sex and FD as covariates. Ms, microstate; DMN, default mode network, **P*<0.05.


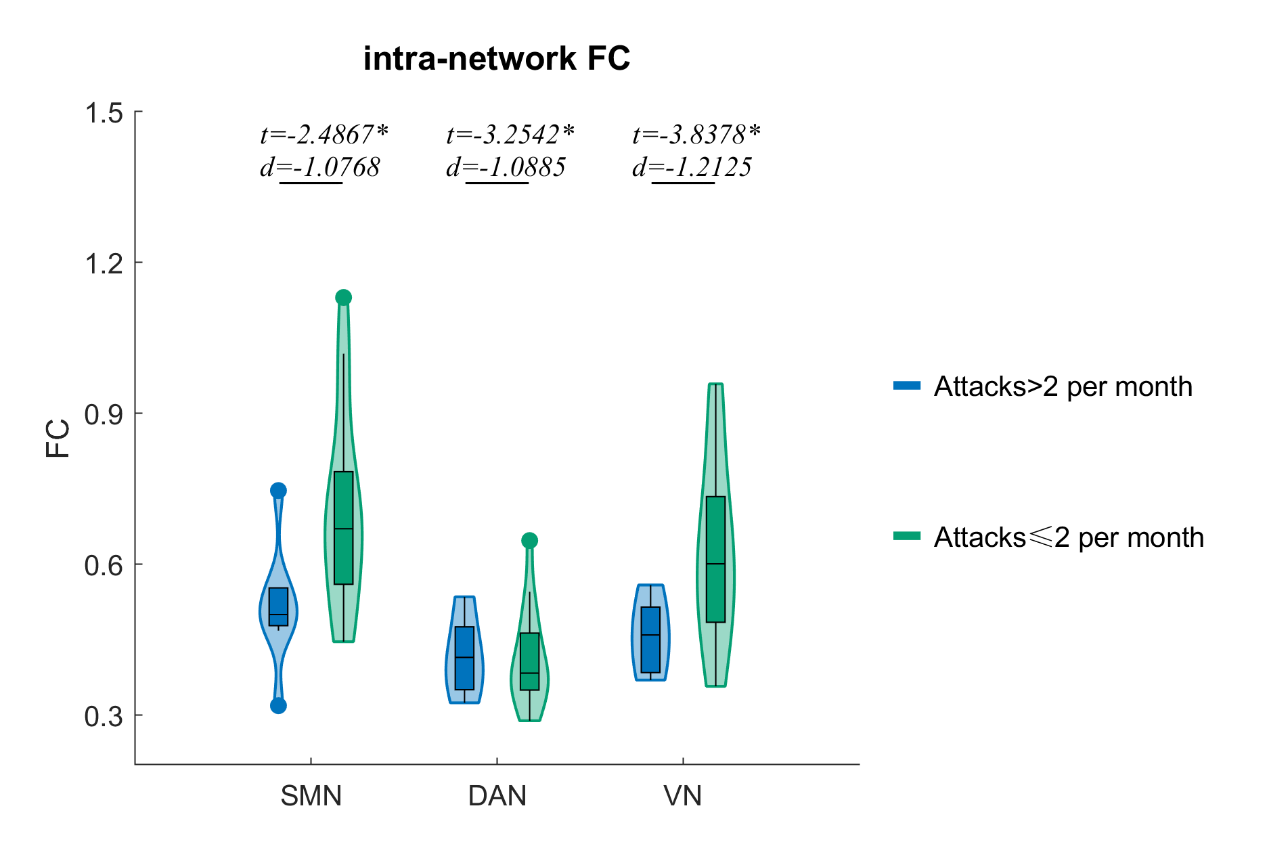


S.Fig.2 Comparison of intra-network FC between subgroups divided by the attack frequency#.

#FC, functional connectivity; SMN, sensory-motor network; DAN, dorsal attention network; VN visual network. *t*, the *t* values of comparisons. *d*, the Cohen’s *d* value. **P*<0.05.


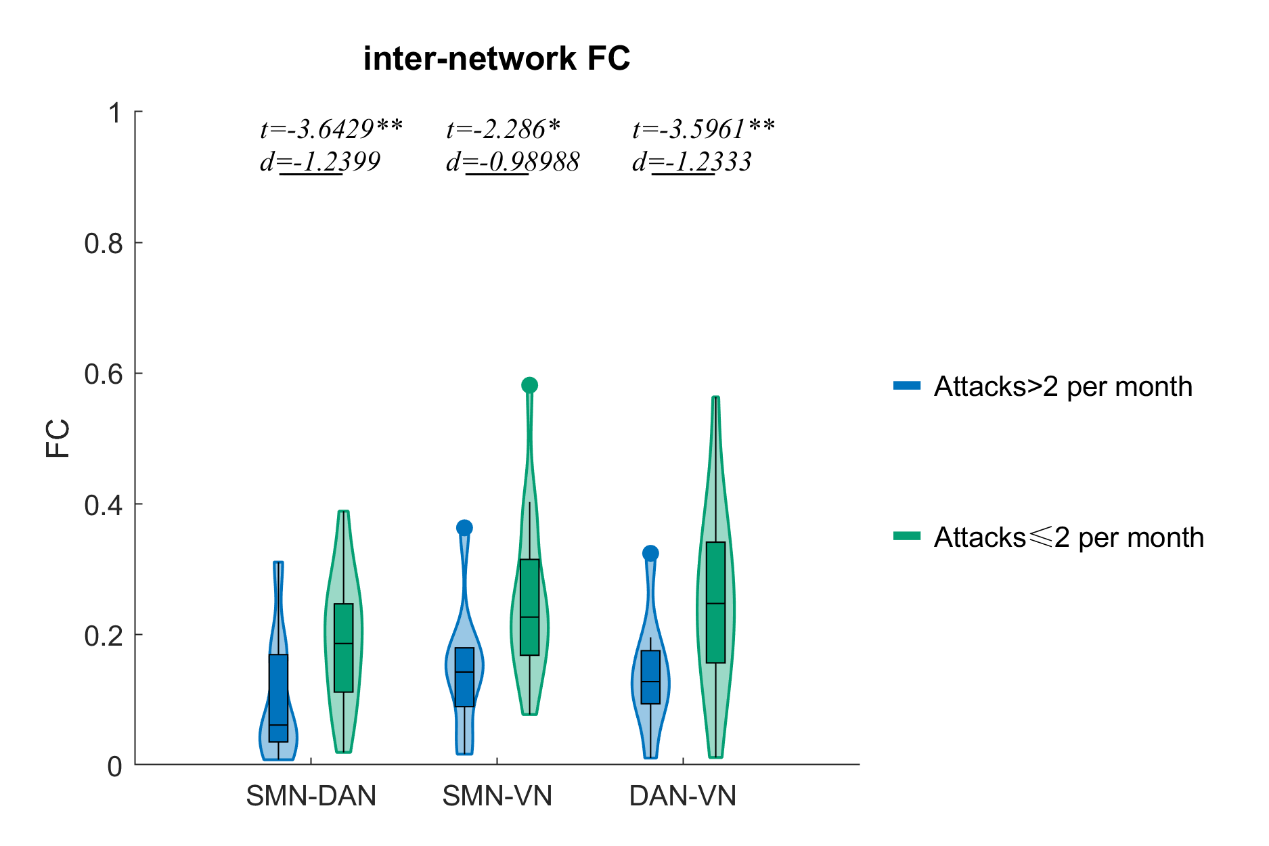


S.Fig.3 Comparison of inter-network FC between subgroups divided by the attack frequency#.

#FC, functional connectivity; SMN, sensory-motor network; DAN, dorsal attention network; VN visual network. *t*, the *t* values of comparisons. *d*, the Cohen’s *d* value. *P<0.05, ***P*<0.01.


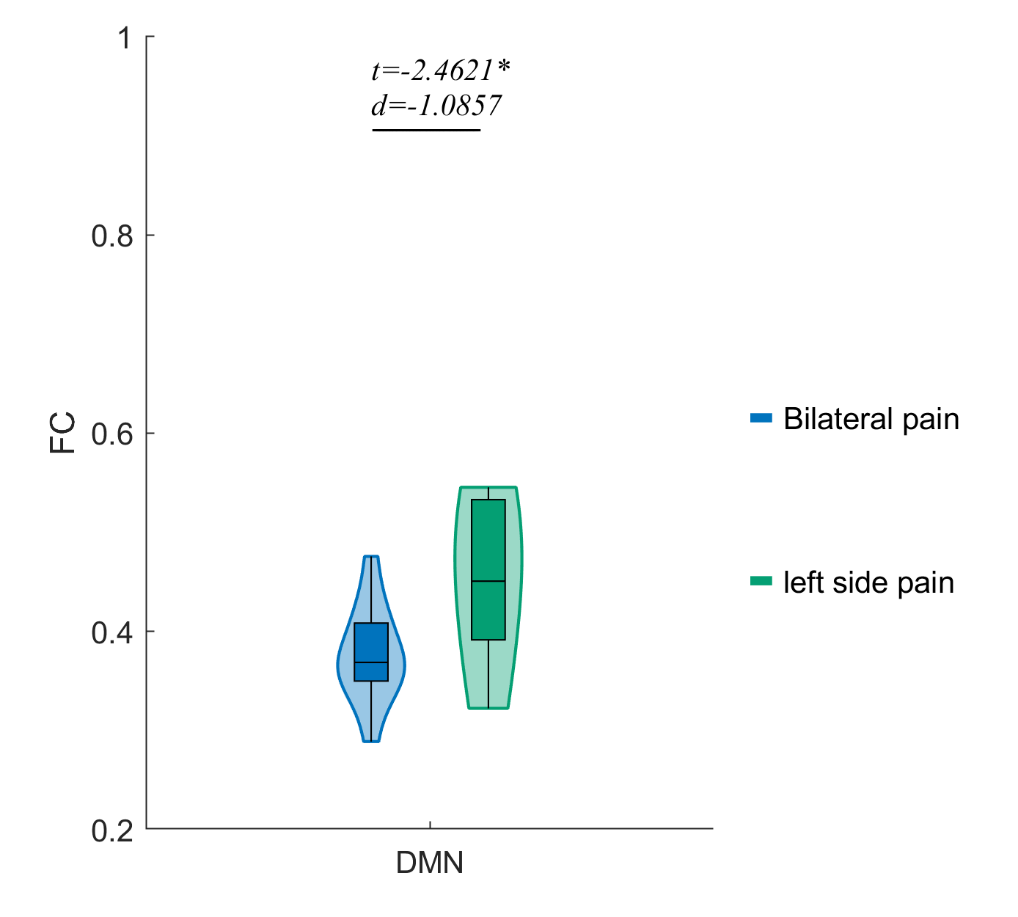


S.Fig.4 Comparison of intra-network FC between subgroups divided by pain sides#.

#FC, functional connectivity; DMN, Default mode network. *t*, the *t* values of comparisons. *d*, the Cohen’s *d* value. **P*<0.05.


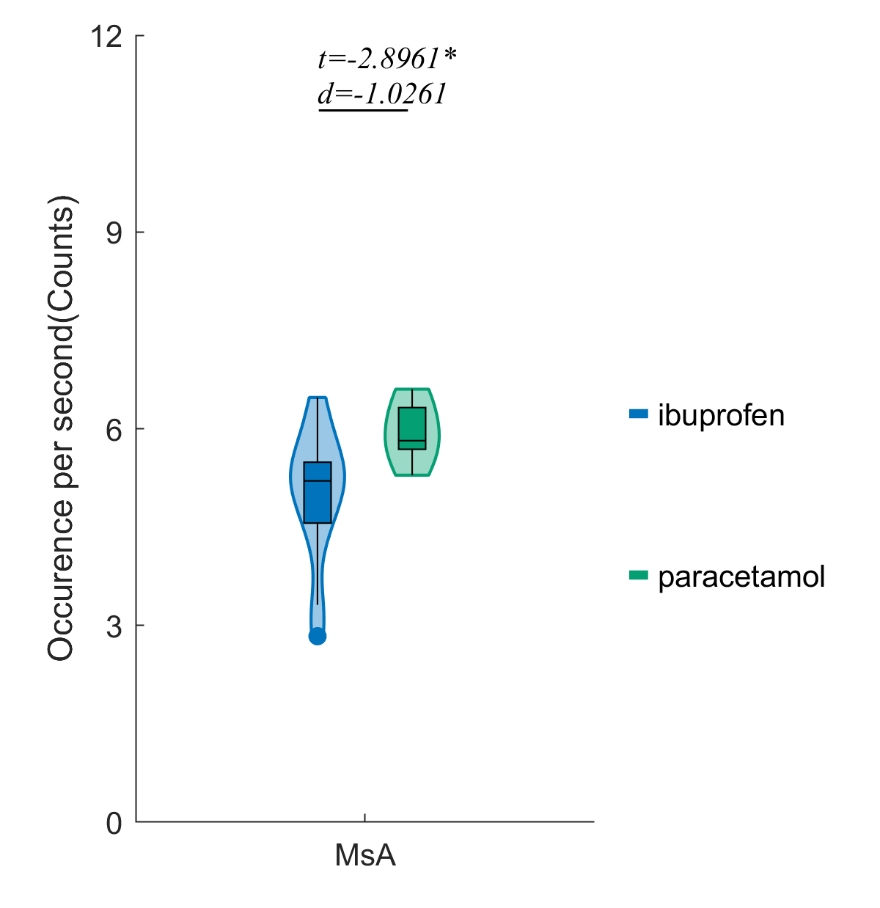


S.Fig.5 Comparison of microstate parameters between subgroups divided by the medications used during attacks#.

#FC, functional connectivity; Ms, microstate. *t*, the *t* values of comparisons. *d*, the Cohen’s *d* value. **P*<0.05.


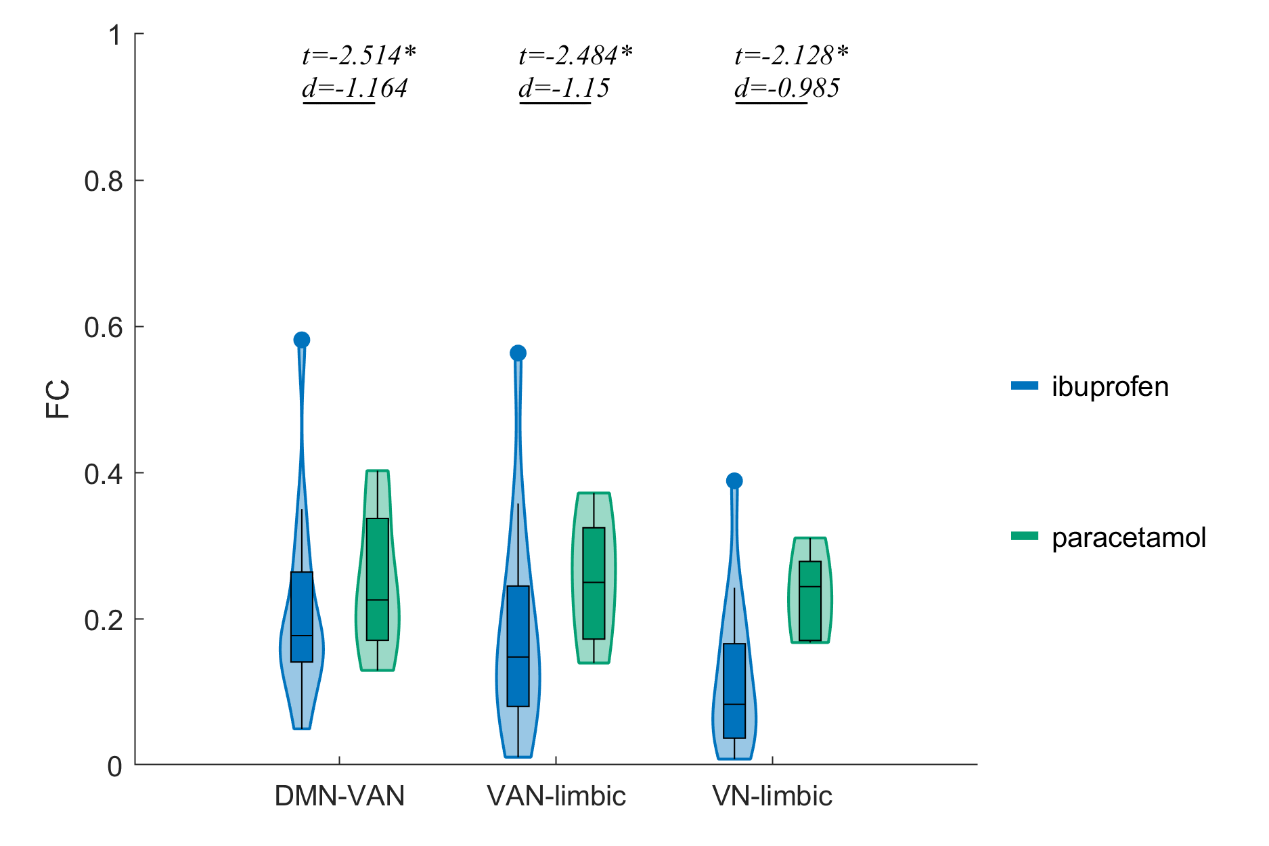


S.Fig.6 Comparison of inter-network FC between subgroups divided by the medications used during attacks#..

#FC, functional connectivity; DMN, Default mode network; VAN,ventral attention network; VN visual network. *t*, the *t* values of comparisons. *d*, the Cohen’s *d* value. **P*<0.05.
